# Photothermal conversion efficiency of saline nanofluids: coupled effects of Fe_3_O_4_ nanoparticle mass fraction and salinity revealed by response surface and random forest modeling

**DOI:** 10.3389/fchem.2026.1890307

**Published:** 2026-07-13

**Authors:** Xiaojun Guo, Jia Liu, Zhiming Wu, Longjie Wang, Da Lin, Wenfeng Xiao, Xiao Song, Qianzhuo Zhu, Yuanrui Zhao, Yichuan Luo, Zhuangzhuang Jia

**Affiliations:** 1 College of Mechanical and Electronic Engineering, Tarim University, Alar, China; 2 College of Engineering, China Agricultural University, Beijing, China; 3 College of Hydraulic and Architectural Engineering, Tarim University, Alar, China

**Keywords:** Fe3O4 nanoparticles, photothermal conversion, random forest regression, response surface methodology, saline nanofluid

## Abstract

Nanofluids have shown great potential for enhancing solar photothermal conversion efficiency, yet most studies have focused on pure-water-based systems, and the coupled effects of nanoparticle mass fraction and salinity remain poorly understood. In this work, Fe_3_O_4_–NaCl nanofluids (0–0.20 wt% Fe_3_O_4_, 0–8 g L^-1^ salinity) were evaluated through outdoor solar-heating and indoor cooling experiments comprising 31 experimental groups. Two-way ANOVA, Pearson correlation analysis, a Ridge-regularized quadratic polynomial model, and random forest (RF) regression with leave-one-out cross-validation (LOOCV) were employed for statistical analysis and prediction. Salinity was identified as the dominant negative factor affecting *η*_nf_ (*p* < 0.001, partial *η* ^2^ = 0.769, *r* = −0.825), whereas nanoparticle mass fraction showed no significant effect (*p* = 0.586). *η*_nf_ exhibited a strong positive correlation with the cooling constant *B* (*r* = 0.866) but only a weak correlation with the maximum temperature rise Δ*T*_max_, indicating that heat dissipation behavior plays a more important role than peak temperature in determining photothermal performance. The optimized RF model outperformed the polynomial model, achieving a LOOCV *R* ^2^ of 0.757 (RMSE = 0.0377, MAE = 0.0301), and feature importance analysis further showed that salinity-related descriptors contributed 71.6% of the total importance. Although the highest predicted *η*_nf_ was obtained under zero-salinity conditions, a practical operating window (2–4 g L^-1^ salinity and 0.05–0.10 wt% Fe_3_O_4_) was identified for applications requiring saline adaptability. These findings provide quantitative insights into the photothermal behavior of saline nanofluids and guidance for their application in solar thermal utilization and low-salinity desalination.

## 1 Introduction

Solar photothermal conversion is one of the key technologies for addressing global energy challenges and achieving carbon neutrality goals (Jin et al., 2025; Wu et al., 2025). The optical absorption and thermal conversion capabilities of the working fluid directly affect the overall system efficiency. Nanofluids, prepared by dispersing nanoscale particles such as metals (Seifikar et al., 2022), metal oxides (Kim et al., 2024), carbon-based materials (George and Sreekanth, 2025), and plasmonic nanostructures (Cardozo et al., 2025; Farooq et al., 2025; Farooq et al., 2026) into base fluids, have attracted considerable attention for improving photothermal conversion efficiency because of their enhanced optical absorption and thermophysical properties (Alsagri and Alrobaian, 2026; Kazaz et al., 2023). Among them, Fe_3_O_4_ nanoparticles have attracted considerable attention in solar desalination (Dong et al., 2025; Zhu et al., 2025) and photothermal therapy (Yang et al., 2025) owing to their efficient photothermal conversion capability, magnetic responsiveness, and chemical stability.

However, most previous studies on nanofluid photothermal conversion have used pure water or ethylene glycol as the base fluid, while the saline environments widely encountered in practical applications have been largely neglected. In practical scenarios such as low-salinity brackish water desalination, the upper low-salinity region of salt-gradient solar ponds (salt concentration: 0–8 g L^-1^), and certain industrial low-salinity wastewater treatments, the working fluid generally contains a certain concentration of dissolved salts. Previous studies have shown that the addition of nanoparticles in applications such as salt-gradient solar ponds (Poyyamozhi et al., 2025), seawater desalination (Rousta et al., 2025) and solar still systems (Chen et al., 2023) can effectively improve the thermal efficiency of the system; however, salt concentration significantly affects the thermophysical properties of nanofluids, thereby altering their heat-transfer enhancement performance (Bretado-de los Rios et al., 2025).

Ions in saline solutions, such as Na^+^ and Cl^−^, can modify the surface charge, electric double-layer structure, and aggregation behavior of nanoparticles (Bohinc et al., 2023), further influencing suspension stability (Chen et al., 2023), optical absorption spectra, and photothermal conversion efficiency (He et al., 2018). Previous studies have shown that nanoparticle stability, particle size, morphology, and aggregation behavior play critical roles in determining the thermal-transport performance of nanofluids. Changes in ionic strength may therefore influence photothermal conversion not only through optical effects but also through modifications of nanoparticle dispersion stability (Farooq et al., 2025). Compared with water-based nanofluids, saline nanofluids exhibit more complex behavior: Moderate salinity may alter nanoparticle dispersion behavior through electric double-layer compression, whereas excessive salinity may induce aggregation and sedimentation (Wu et al., 2022). Therefore, it is necessary to systematically investigate the coupled effects of salt concentration and nanoparticle mass fraction on photothermal conversion performance.

In terms of research methodology, data-driven models such as response surface methodology (RSM), deep forest (DF), and random forest (RF) have recently been applied to predict the thermophysical properties of nanofluids, including thermal conductivity and viscosity, and have achieved promising results (Cai et al., 2025; Eshgarf et al., 2023; He et al., 2022; Kamsuwan et al., 2025; Naseri et al., 2022; Ren et al., 2024). Recent studies have further demonstrated the applicability of machine-learning techniques in thermal-fluid systems. For example, machine-learning-assisted prediction has been successfully applied to ferro-nanofluid flow behavior, magnetohydrodynamic heat-transfer processes, and nanofluid-based battery cooling systems, highlighting the capability of data-driven approaches to capture complex nonlinear relationships in heat-transfer phenomena (Ibrahim et al., 2026; Sharma et al., 2026; Shukla et al., 2026). Nevertheless, these models have primarily been developed for water-based nanofluids and focus on thermophysical properties rather than photothermal conversion efficiency. In particular, the interacting effects of nanoparticle mass fraction and salt concentration—a critical factor in saline environments—have not been incorporated into existing predictive frameworks. Consequently, quantitative analysis and data-driven optimization of photothermal efficiency under saline conditions remain largely unexplored.

Therefore, Fe_3_O_4_ nanoparticle–NaCl solution–water was selected as the model system in this study to systematically investigate the coupled effects of nanoparticle mass fraction (0%–0.20%) and salt concentration (0–8 g L^-1^) on the photothermal conversion performance of saline nanofluids. Temperature-response and irradiation-intensity data of 31 samples were obtained through outdoor solar-heating and indoor cooling experiments, and the photothermal conversion efficiency of the nanofluids was subsequently calculated. Quadratic polynomial response surface modeling with Ridge regularization and feature-engineered random forest (RF) modeling were employed to establish predictive models. Leave-one-out cross-validation (LOOCV) was used to evaluate model performance and identify the optimal predictive framework. This study aims to reveal the influence of salt concentration and nanoparticle mass fraction on photothermal conversion efficiency and to provide experimental insight and data-driven analysis for understanding the photothermal conversion behavior of saline nanofluids in solar thermal applications.

## 2 Materials and methods

### 2.1 Materials

Fe_3_O_4_ nanoparticles were purchased from Shanghai Yaoyi Co., Ltd., with an average particle size of 103 nm. The SEM/TEM images and particle size distribution are presented in Supplementary Figure S1. Sodium chloride (NaCl, analytical grade, purity ≥ 99.5%) was used as the salt source. Deionized water was employed as the base fluid. All reagents were used as received without further purification.

### 2.2 Preparation of saline nanofluids

**FIGURE 1:**
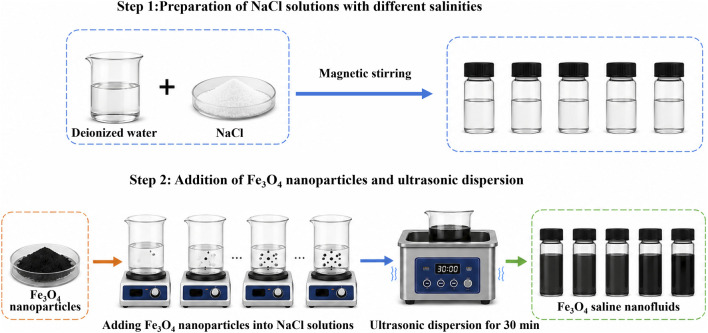
Schematic illustration of the two-step preparation process of Fe_3_O_4_ saline nanofluids.

Saline nanofluids were prepared using a conventional two-step dispersion method (Wang et al., 2023). First, different amounts of NaCl were dissolved in deionized water under magnetic stirring to prepare saline solutions with salt concentrations of 0, 2, 4, 6, and 8 g L^-1^. Subsequently, Fe_3_O_4_ nanoparticles were added into the saline solutions at mass fractions of 0.02%, 0.04%, 0.06%, 0.08%, 0.10%, and 0.20%, respectively. The suspensions were then ultrasonically dispersed for 30 min to obtain uniformly dispersed Fe_3_O_4_ saline nanofluids. Pure water without nanoparticles (nanoparticle mass fraction: 0%; salt concentration: 0 g L^-1^) was used as the CK control group. To minimize nanoparticle sedimentation and ensure dispersion stability, all photothermal conversion experiments were conducted immediately after preparation. A schematic illustration of the preparation process of the Fe_3_O_4_ saline nanofluids is shown in Figure 1.

### 2.3 Experimental setup for photothermal conversion

**FIGURE 2:**
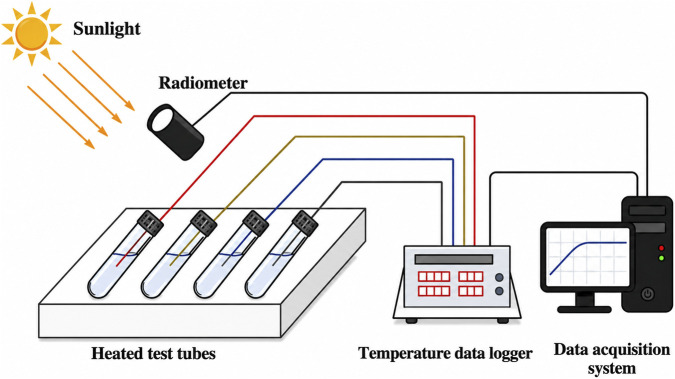
Experimental setup for photothermal conversion measurements.

The photothermal conversion experimental setup is illustrated in Figure 2. The system mainly consisted of glass test tubes (inner diameter: 12 mm; length: 53 mm), an inclined support plate adjusted to maintain approximate perpendicularity between the test tubes and incident sunlight, a solar irradiance meter (measurement range: 0–2000 W·m^-2^; accuracy: ±10%), a temperature acquisition system (accuracy: ±0.5 °C), and a computer for data acquisition.

To minimize heat loss, the bottom and side surfaces of the glass test tubes were wrapped with 20 mm-thick thermal insulation cotton, leaving only the upper exposed region for maximum solar absorption. The sensing surface of the irradiance meter was oriented toward the light source and positioned perpendicular to the test tubes to ensure that the measured irradiance accurately represented the actual radiation intensity received by the illuminated surface of the test tubes.

### 2.4 Experimental procedure

Before the experiments, clear weather conditions and stable solar irradiation were ensured, with irradiance fluctuations controlled within 10%. The photothermal conversion experiments consisted of an outdoor heating stage followed by an indoor cooling stage.

1. Heating stage. Prepared saline nanofluids were injected into glass test tubes indoors, sealed with rubber stoppers, and equipped with thermocouples inserted into the liquid phase. The test tubes were then placed at their designated positions. After the temperatures of all groups became stable, the tubes were transferred outdoors under direct sunlight exposure. From the beginning of the experiment (defined as 0 min), nanofluid temperatures were recorded every 30 s, while solar irradiance was recorded every 5 min. Heating continued until thermal equilibrium was reached, which required approximately 90 min.
2. Cooling stage. After the heating stage, the test tubes were rapidly transferred from outdoors to indoors without direct sunlight exposure (ambient temperature: 25 °C ± 1 °C) to initiate the cooling experiments. Temperature data were recorded every 30 s until the nanofluid temperature approached ambient temperature, which required approximately 90 min.

A total of 31 experimental groups, including the CK group, were investigated. Pure water was used as the CK control group. The experimental design matrix is summarized in Table 1.

**TABLE 1:** Experimental design matrix and sample groups for photothermal conversion experiments of saline nanofluids.

Fe_3_O_4_ nanoparticle mass fraction (%)	Salt concentration (g·L^-1^)				
	0g·L^–1^ NaCl	2g·L^–1^ NaCl	4g·L^–1^ NaCl	6g·L^–1^ NaCl	8g·L^–1^ NaCl
—	0	2	4	6	8
0.02	1	7	13	19	25
0.04	2	8	14	20	26
0.06	3	9	15	21	27
0.08	4	10	16	22	28
0.10	5	11	17	23	29
0.20	6	12	18	24	30

Pure water without nanoparticles (nanoparticle mass fraction: 0%; salt concentration: 0 g·L^-1^) was used as the CK control group.

### 2.5 Calculation of performance indicators

#### 2.5.1 Specific heat capacity

The specific heat capacity of the saline nanofluids was calculated according to the mixture rule (Murshed, 2012), as shown in Equation 1.

$$c_{p}=\frac{w_{np}}{100}c_{p,\text{np}}+\frac{w_{salt}}{100}c_{p,\text{salt}}+\left(1‐\frac{w_{\text{np}}}{100}‐\frac{w_{\text{salt}}}{100}\right)c_{p,\text{water}}$$ (1)

Where *w*_np_ and *w*_salt_ are the mass fractions (%) of Fe_3_O_4_ nanoparticles and NaCl, respectively; *c*_*p*,np_, *c*_*p*,salt_ and *c*_*p*,water_ were taken as 0.86, 0.86, and 4.18 kJ kg^-1^·K^−1^, respectively.

#### 2.5.2 Heat dissipation coefficient *B*

During the cooling stage, the variation in nanofluid temperature with time followed Newton’s law of cooling, as shown in Equation 2:

$$\ln\left[\frac{T\left(t\right)‐T_{\text{amb}}}{T_{0}‐T_{\text{amb}}}\right]=‐Bt$$ (2)

where *T*(*t*) is the temperature at time *t*,*T*_amb_ is the ambient temperature, taken as the average indoor temperature of 25 °C, and *T*_0_ is the initial cooling temperature after the sample was transferred indoors. The heat dissipation coefficient *B* (s^-1^) was obtained from the slope of the linear fitting between ln ((*T*−*T*_amb_)/(*T*_0_−*T*_amb_)) and time *t.*

#### 2.5.3 Photothermal conversion efficiency of nanofluids

**FIGURE 3:**
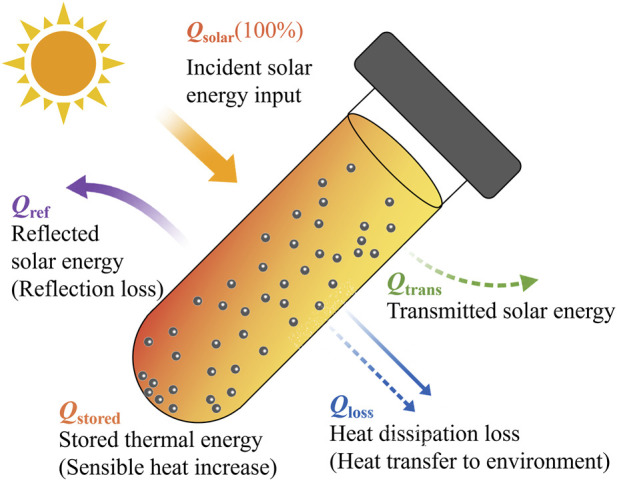
Schematic illustration of energy balance pathways during photothermal conversion of saline nanofluids under solar irradiation.

To further interpret the heat transfer behavior during photothermal conversion, an energy balance framework was employed to analyze the distribution of incident solar energy in the sealed saline nanofluid system. As illustrated in Figure 3, the incident solar energy was partitioned into stored thermal energy, reflected energy, and heat dissipation loss to the surrounding environment. The stored thermal energy was mainly associated with the sensible heat increase of the nanofluid, whereas heat dissipation included convective, conductive, and radiative heat transfer processes.

The photothermal conversion efficiency of the nanofluids (*η*_nf_) was defined as the fraction of incident solar energy effectively stored as sensible heat in the nanofluid and was calculated as shown in Equation 3:

$$\eta_{nf}=\frac{mc_{p}\Delta T_{\max}}{A\int G\left(1‐R\right)\;dt}$$ (3)

Where *m* is the mass of the nanofluid (kg), *c*_*p*_ is the specific heat capacity (J·kg^-1^·K^−1^), Δ*T*_max_ is the maximum temperature rise during the heating stage, defined as the difference between the equilibrium temperature and the initial temperature ( °C), *A* is the illuminated area of the test tube (m^2^), *G* is the measured solar irradiance (W·m^-2^), and *R* is the reflectance of the test tube, with *R* = 0.1. The integration time ranged from 0 to the equilibrium time. The illuminated area was calculated based on the cylindrical side area of the exposed part of the test tube, with *A*≈0.001 m^2^.

#### 2.5.4 System photothermal conversion efficiency

The system photothermal conversion efficiency (*η*_sys_) was used to evaluate the cumulative energy conversion over the entire heating process, as shown in Equation 4:

$$\eta_{\text{sys}}=\frac{\sum_{i}mc_{p}\Delta T_{i}}{A\sum_{i}G_{i}\Delta t}$$ (4)

where Δ*T*_*i*_ is the temperature rise during the *i*-th time interval ( °C), *G*_*i*_ is the average solar irradiance during the corresponding interval (W·m^-2^), and Δ*t* = 30s is the sampling interval. The numerator represents the cumulative heat absorbed by the nanofluid, while the denominator represents the total incident solar energy.

### 2.6 Data analysis methods

#### 2.6.1 Response surface methodology

A quadratic polynomial model within the RSM framework was employed to fit the relationship between nanofluid photothermal conversion efficiency *y*, nanoparticle mass fraction *x*_1_ (%), and salt concentration *x*_2_ (g·L^-1^), as shown in Equation 5:

$$y=\beta_{0}+\beta_{1}x_{1}+\beta_{2}x_{2}+\beta_{11}x_{1}^{2}+\beta_{22}x_{2}^{2}+\beta_{12}x_{1}x_{2}+\varepsilon$$ (5)

The regression coefficients were estimated using the least-squares method. To avoid unstable coefficient estimation caused by multicollinearity among variables, Ridge regularization (L2 penalty) was introduced, and the objective function was defined as shown in Equation 6:

$$\min_{\beta }\left\{\sum_{i=1}^{n}{\left(y_{i}‐{\hat{y}}_{i}\right)}^{2}+\alpha \sum_{j=1}^{p}\beta_{j}^{2}\right\}$$ (6)

where *α* is the regularization strength parameter. The optimal *α* value was selected from the set {0.01,0.1,1,10} using five-fold cross-validation to minimize the prediction error. Model fitting was implemented using the RidgeCV algorithm in the scikit-learn library.

#### 2.6.2 Random forest model

RF is an ensemble learning method that improves prediction accuracy and reduces overfitting by constructing multiple decision trees and averaging their prediction results (Breiman, 2001). In this study, a random forest regression model was established with *x*_1_ and *x*_2_ as input features and *η*_nf_ as the output variable. Additional nonlinear and interaction descriptors derived from *x*_1_ and *x*_2_ were incorporated into the model to improve its ability to capture nonlinear relationships between operating variables and *η*_nf_. The RF model was implemented using the RandomForestRegressor algorithm in the scikit-learn library.

To reduce the risk of overfitting under the limited-sample condition, all model selection and performance evaluation procedures were conducted using leave-one-out cross-validation (LOOCV). Hyperparameter optimization was performed by adjusting the number of trees (*n_estimators*), maximum tree depth (*max_depth*), minimum samples required for node splitting (*min_samples_split*), minimum samples per leaf (*min_samples_leaf*), and the proportion of features used for node splitting (*max_features*). The parameter combination yielding the highest LOOCV *R* ^2^ value was selected as the final model.

The optimized RF model parameters were *n_estimators* = 796, *max_depth* = 12, *min_samples_split* = 2, *min_samples_leaf* = 2, *max_features* = 0.322, *bootstrap* = False, and *random_state* = 168.

Feature importance was evaluated using impurity-based feature importance, which quantifies the relative contribution of each descriptor to reducing prediction error in the RF model.

#### 2.6.3 Model evaluation methods

Due to the relatively small sample size (*n* = 31), LOOCV was employed to evaluate the generalization capability of the models (Geroldinger et al., 2023). In LOOCV, one sample was retained as the test set in each iteration, while the remaining *n*−1 samples were used as the training set. This process was repeated *n* times to obtain *n* predicted values. The following two evaluation metrics were calculated using Equations 7, 8, respectively:

Root Mean Square Error (RMSE):

$$\text{RMSE}=\sqrt{\frac{1}{n}\sum_{i=1}^{n}{\left(y_{i}‐{\hat{y}}_{i}\right)}^{2}}$$ (7)

Coefficient of Determination (*R* ^2^):

$$R^{2}=1‐\frac{\sum_{i=1}^{n}{\left(y_{i}‐{\hat{y}}_{i}\right)}^{2}}{\sum_{i=1}^{n}{\left(y_{i}‐\bar{y}\right)}^{2}}$$ (8)

Where ${\hat{y}}_{i}$ is the predicted value of the *i*-th sample, and $\bar{y}$ is the mean value of the experimental data. A smaller RMSE and an *R* ^2^ value closer to 1 indicate better predictive performance of the model.

#### 2.6.4 Correlation analysis

To evaluate the degree of linear correlation, the Pearson correlation coefficient *r* between all continuous variables was calculated (Tang et al., 2023), as shown in Equation 9:

$$r=\frac{\sum_{i=1}^{n}\left(x_{i}‐\bar{x}\right)\left(y_{i}‐\bar{y}\right)}{\sqrt{\sum_{i=1}^{n}{\left(x_{i}‐\bar{x}\right)}^{2}}\sqrt{\sum_{i=1}^{n}{\left(y_{i}‐\bar{y}\right)}^{2}}}$$ (9)

Correlation coefficients with |*r*| ≥0.8 were considered strong correlations, 0.5≤ |*r*| <0.8 indicated moderate correlations, 0.3≤ |*r*| <0.5 indicated weak correlations, and |*r*| <0.3 indicated no significant correlation.

#### 2.6.5 Software and tools

All data analyses were conducted using Python 3.9. The main libraries employed included NumPy for numerical computation, pandas for data processing, scikit-learn for machine learning and cross-validation, and matplotlib and seaborn for visualization.

To facilitate comparison between the two predictive models used in this study, their main characteristics, advantages, and limitations are summarized in Table 2.

**TABLE 2:** Comparison of the characteristics, advantages, and limitations of the two predictive models used in this study.

Model	Main characteristics	Advantages	Limitations
Quadratic polynomial model with ridge regularization	Explicit second-order regression model	Interpretable equation; suitable for engineering calculations	Limited ability to describe complex nonlinear relationships
Random forest (RF)	Ensemble learning based on decision trees	Robust prediction and feature importance analysis	Model interpretation is less straightforward than regression-based models; performance may be limited for small datasets

## 3 Results and discussion

### 3.1 Irradiation conditions and typical temperature response

**FIGURE 4:**
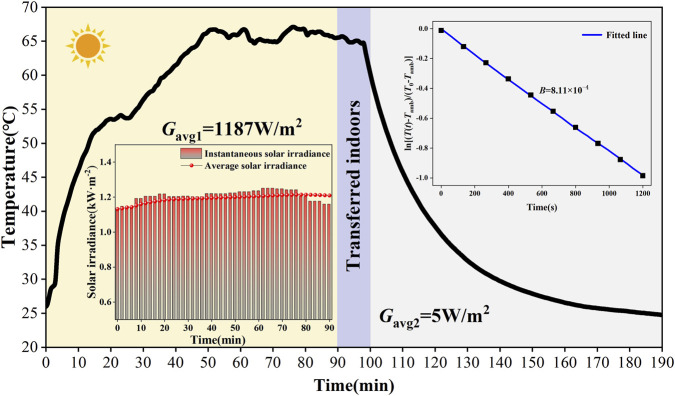
Variation in solar irradiance and temperature response of the pure water group.

The variation in solar irradiance during the experiments and the temperature response of the pure water group are shown in Figure 4.

The solar irradiance was approximately 1130 W m^-2^ during the initial stage of the experiment (0–15 min), gradually increased to approximately 1250 W m^-2^, and then slightly decreased during the later stage (after 80 min). The average solar irradiance during the entire heating stage was 1187 ± 52 W m^−2^, with fluctuations below 5%, indicating stable weather conditions and satisfying the steady-state analysis assumption.

During the heating stage (0–90 min), the water temperature increased rapidly from 26 °C, and the heating rate gradually decreased after approximately 60 min. Thermal equilibrium was reached at 80 min with an equilibrium temperature of 66.96 °C, corresponding to a maximum temperature rise of 41.14 °C. The period from 90 to 100 min corresponded to the transition from outdoor heating experiments to indoor cooling experiments, and the data during this interval were excluded from subsequent analysis. The cooling stage started at 100 min, during which the temperature exhibited exponential decay behavior. Fitting based on Newton’s law of cooling yielded a heat dissipation coefficient of *B* = 8.11 × 10^−4^ s^−1^ with *R* ^2^ = 0.996. The rapid temperature increase during the initial heating stage suggests efficient solar-energy absorption and photothermal conversion. As the temperature increased, the heating rate gradually decreased because heat losses to the surrounding environment increased, leading to the establishment of thermal equilibrium. The high fitting coefficient (*R* ^2^ = 0.996) obtained from Newtonian cooling analysis indicates that heat dissipation behavior can be effectively characterized using the heat dissipation coefficient *B*, which later showed a strong correlation with *η*_nf_. Representative heating and cooling curves of saline nanofluids under different nanoparticle mass fractions and salt concentrations are provided in Supplementary Figure S2.

### 3.2 Summary of experimental results

The photothermal conversion experimental results of saline nanofluids are summarized in Table 3.

**TABLE 3:** Experimental results of photothermal conversion for saline nanofluids.

Group	*x*_1_	*x*_2_	*η*_nf_	*η*_sys_	Δ*T*_max_ (°C)	*B* (×10^-4^ s^-1^)
CK	0	0	0.76	0.38	41.14	8.11
1	0.02	0	0.79	0.35	40.7	8.36
2	0.04	0	0.8	0.34	41.2	8.4
3	0.06	0	0.82	0.3	41.66	8.5
4	0.08	0	0.8	0.34	45.03	8.07
5	0.1	0	0.73	0.29	36.99	8.54
6	0.2	0	0.77	0.29	38.33	8.8
7	0.02	2	0.69	0.38	42.47	7.11
8	0.04	2	0.67	0.37	42.1	6.9
9	0.06	2	0.67	0.37	43.55	6.73
10	0.08	2	0.69	0.37	41.58	7.13
11	0.1	2	0.73	0.36	39.14	8.26
12	0.2	2	0.78	0.38	40.99	8.18
13	0.02	4	0.69	0.34	39.14	7.52
14	0.04	4	0.67	0.3	34.59	8.38
15	0.06	4	0.7	0.34	38.25	8.16
16	0.08	4	0.64	0.34	39.43	7.03
17	0.1	4	0.65	0.36	39.95	7.17
18	0.2	4	0.62	0.38	43.45	6.64
19	0.02	6	0.58	0.36	42.35	6.29
20	0.04	6	0.6	0.36	43.64	6.12
21	0.06	6	0.54	0.33	41.44	5.79
22	0.08	6	0.66	0.38	41.04	7.33
23	0.1	6	0.61	0.34	40.84	6.64
24	0.2	6	0.71	0.36	40.26	7.63
25	0.02	8	0.61	0.34	38.63	7.29
26	0.04	8	0.64	0.35	41.33	7.26
27	0.06	8	0.56	0.33	38.71	6.68
28	0.08	8	0.58	0.35	39.19	7.04
29	0.1	8	0.6	0.36	41.58	6.83
30	0.2	8	0.67	0.36	41.22	7.46

### 3.3 Analysis of factors influencing nanofluid photothermal conversion efficiency

To quantitatively evaluate the main effects of nanoparticle mass fraction (*x*_1_) and salt concentration (*x*_2_) on the photothermal conversion efficiency of nanofluids (*η*_nf_), two-way analysis of variance (ANOVA, main-effect model) was employed (Sreekumar et al., 2024). The results are presented in Table 4.

**TABLE 4:** Results of two-way ANOVA for nanofluid photothermal conversion efficiency (main-effect model).

Source of variation	Sum of squares (SS)	Degrees of freedom (df)	Mean square (MS)	*F*-value	*p*-value	Partial *η* ^2^
*x*_1_	0.0090	6	0.0015	0.793	0.586	0.191
*x*_2_	0.1268	4	0.0317	16.68	<0.001	0.769
Residual	0.038	20	0.0019	–	–	–

The two-way ANOVA, results indicated that salt concentration exerted a statistically significant effect on *η*_nf_ (*F* = 16.68, *p* < 0.001), with a partial *η* ^2^ value of 0.769, suggesting that salinity was the dominant governing factor affecting photothermal conversion performance. In contrast, nanoparticle mass fraction showed no statistically significant effect within the investigated range (*F* = 0.793, *p* = 0.586), with a partial *η* ^2^ value of 0.191.

Tukey’s honestly significant difference (HSD) *post hoc* test was further employed to perform pairwise comparisons among different salt concentration levels (Zhang et al., 2016), and the results are summarized in Table 5. Compared with 0 g L^-1^, the *η*_nf_ values differed significantly at salt concentrations of 2, 4, 6 and 8 g L^-1^ (*p* < 0.05). Significant differences were also noted between 2 g L^-1^ and 6 g L^-1^ (*p* = 0.0099), and between 2 g L^-1^ and 8 g L^-1^ (*p* = 0.0074), while no statistical significance was observed among the rest groups (*p* > 0.05).

**TABLE 5:** Results of Tukey’s HSD *post hoc* test among different salt concentration levels (only significant pairs are listed).

Comparison group (g·L^-1^)	Mean difference	*p*-value	95% confidence interval
0 vs. 2	−0.0797	0.0184	[-0.1490, −0.0104]
0 vs. 4	−0.1227	<0.001	[-0.1920, −0.0534]
0 vs. 6	−0.1687	<0.001	[-0.2380, −0.0994]
0 vs. 8	−0.1716	<0.001	[-0.2409, −0.1024]
2 vs. 6	−0.0890	0.0099	[-0.1609, −0.0171]
2 vs. 8	−0.0920	0.0074	[-0.1639, −0.0201]

The above analysis indicates that salt concentration was the dominant factor governing the photothermal conversion efficiency of the nanofluids, whereas variations in nanoparticle mass fraction within the range of 0%–0.20% did not result in statistically significant changes in efficiency. Therefore, the subsequent modeling analysis focused solely on *η*_nf_.

### 3.4 Predictive modeling of nanofluid photothermal conversion efficiency

#### 3.4.1 Correlation analysis of variables

**FIGURE 5:**
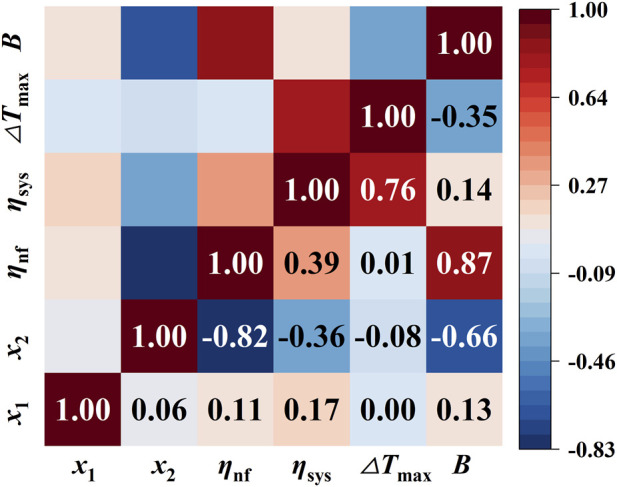
Pearson correlation coefficient heatmap of all variables.

Figure 5 presents the Pearson correlation heatmap among the investigated variables. The photothermal conversion efficiency of saline nanofluids (*η*_nf_) exhibited a strong negative correlation with salt concentration (*x*_2_, *r* = −0.825) and only a weak positive correlation with nanoparticle mass fraction (*x*_1_, *r* = 0.108), further confirming that salinity was the dominant factor governing *η*_nf_. In contrast, *η*_nf_ showed a strong positive correlation with the heat dissipation coefficient *B* (*r* = 0.866), whereas almost no correlation was observed between *η*_nf_ and the maximum temperature rise (Δ*T*_max_, *r* = 0.014). These results suggest that heat dissipation behavior showed a stronger correlation with *η*_nf_ than peak temperature in determining photothermal conversion performance. In addition, *B* exhibited a moderate negative correlation with salt concentration (*r* = −0.664), indicating that increasing salinity significantly altered the heat dissipation behavior of the nanofluid system.

#### 3.4.2 Quadratic polynomial response surface model

Based on the experimental data, a quadratic polynomial model was established using Ridge regularization with *α* = 0.01. The leave-one-out cross-validation (LOOCV) results yielded *R* ^2^ = 0.663 and RMSE = 0.0443, indicating acceptable predictive capability under limited-sample conditions. The regression equation is expressed as shown in Equation 10:

$$\eta_{nf}=0.7983‐0.4172x_{1}‐0.046x_{2}+2.0879x_{1}^{2}+0.00259x_{2}^{2}+0.0461x_{1}x_{2}$$ (10)

**FIGURE 6:**
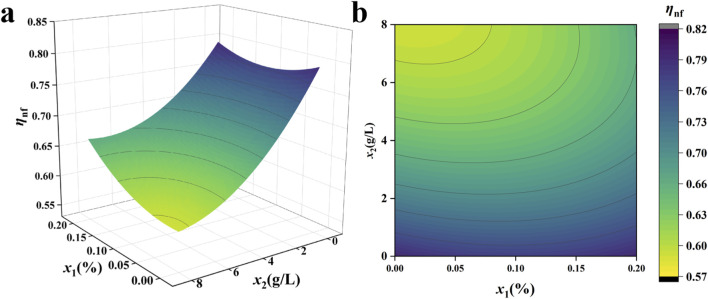
Quadratic polynomial response surface model: **(a)** 3D response surface plot; **(b)** contour plot.

Figure 6 presents the response surface (3D) and contour plots of the quadratic polynomial model with Ridge regularization. As shown in Figure 6a, *η*_nf_ decreased significantly with increasing salt concentration (*x*_2_), whereas its variation with nanoparticle mass fraction (*x*_1_) remained relatively moderate. The contour plot in Figure 6b further indicates that relatively high *η*_nf_ values were mainly concentrated in the low-salinity region (0–2 g L^-1^). In comparison, the influence of nanoparticle mass fraction on *η*_nf_ was considerably weaker within the investigated range. The response surface further suggests that the beneficial effect of increasing nanoparticle concentration was limited compared with the detrimental effect of salinity. Although higher nanoparticle concentrations provide additional light-absorbing centers, increasing salinity may simultaneously reduce nanoparticle utilization efficiency through aggregation and stability deterioration. Consequently, the interaction between nanoparticle concentration and salinity was dominated by the influence of salt concentration. This phenomenon may be partially attributed to salinity-induced aggregation of Fe_3_O_4_ nanoparticles. Increasing ionic strength can compress the electrical double layer surrounding nanoparticles, thereby reducing electrostatic repulsion and promoting particle aggregation. Such aggregation may decrease nanoparticle dispersion stability and reduce the effective utilization of dispersed nanoparticles, which could further weaken the positive contribution of nanoparticle loading to photothermal conversion performance (Bohinc et al., 2023; Farooq et al., 2025).

Overall, the response surface model reasonably described the variation trend of *η*_nf_ under different operating conditions and provided an explicit mathematical relationship between the investigated variables and photothermal conversion efficiency.

#### 3.4.3 Random forest model

**FIGURE 7:**
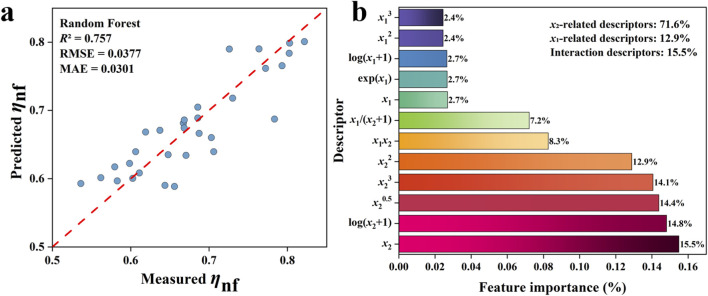
Performance and interpretation of the RF model: **(a)** predicted versus measured *η*_nf_ values; **(b)** feature importance of the RF model.

The optimized RF model showed satisfactory predictive capability for *η*_nf_ prediction under limited-sample conditions. The model achieved an LOOCV performance of *R* ^2^ = 0.757, RMSE = 0.0377, and MAE = 0.0301, outperforming the quadratic polynomial model. Figure 7a presents the comparison between measured and predicted values. Although deviations from the ideal parity line were observed for several samples, the RF model successfully captured the overall variation trend of *η*_nf_.

It should be noted that the present dataset consisted of only 31 experimental samples, which inherently limits the achievable prediction accuracy of data-driven models. Similar machine-learning studies in materials synthesis have frequently been developed using experimental datasets containing only several tens of samples. For example, Williams et al. (2023) developed random-forest models based on 31 experimental runs, while Pellegrino et al. (2020) established ANN models using 20–34 hydrothermal synthesis experiments. Therefore, despite the limited sample size, the obtained LOOCV results suggest that the RF model successfully captured the dominant relationships between operating variables and *η*_nf_ while maintaining acceptable generalization ability.

Feature importance analysis indicated that salinity-related descriptors collectively dominated the prediction outcome (Figure 7b). The five most influential descriptors were *x*_2_, log (*x*_2_+1), *x*_2_^0.5^, *x*_2_^3^, and *x*_2_^2^, all of which were associated with salinity. Collectively, salinity-related descriptors contributed approximately 71.6% of the total importance, whereas nanoparticle-related descriptors contributed only 12.9%. Interaction descriptors accounted for the remaining 15.5%. This result further confirms that salt concentration remained the primary governing factor affecting *η*_nf_ throughout the investigated experimental range.

This finding is also consistent with previous studies. Salinity-induced changes in nanoparticle dispersion stability and aggregation behavior may significantly influence the photothermal performance of nanofluids (Farooq et al., 2025).

**FIGURE 8:**
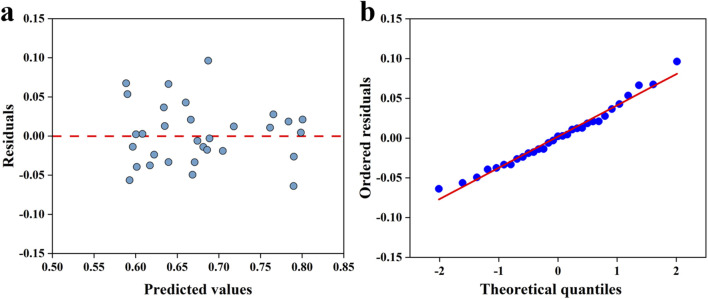
Residual analysis of the RF model: **(a)** scatter plot of residuals versus predicted values; **(b)** Q–Q plot.

Residual analysis showed that the residuals were generally symmetrically distributed around zero without obvious systematic patterns, as illustrated in Figure 8. In addition, the Q–Q plot showed that most residual points approximately followed the reference line, indicating acceptable prediction stability of the RF model under limited-sample conditions.

The RF model further confirmed that salinity was the dominant factor governing *η*_nf_ variation. Model prediction results indicated that relatively high photothermal conversion efficiencies were mainly concentrated in the low-salinity region. The addition of salt ions may alter the electric double-layer structure and aggregation behavior of nanoparticles, thereby affecting suspension stability and photothermal conversion performance (Bohinc et al., 2023). However, in practical applications such as solar seawater desalination, saline environments are unavoidable. Therefore, identifying relatively high-efficiency operating regions under saline conditions remains practically important. The model prediction results showed that the highest predicted *η*_nf_ appeared under zero-salinity conditions at a relatively low nanoparticle mass fraction, which further supports the suppressive effect of salinity on photothermal conversion performance. Nevertheless, considering that saline conditions are unavoidable in practical applications, the prediction results also suggested that relatively high *η*_nf_ values could still be maintained under moderate salinity conditions when suitable nanoparticle mass fractions were employed. Therefore, the range of approximately 2–4 g L^-1^ salinity with 0.05%–0.10% Fe_3_O_4_ may be regarded as a practical operating region that balances saline adaptability and photothermal conversion performance.

A further limitation of the present study is that nanoparticle aggregation under saline conditions was not directly characterized in the present study. Therefore, the observed reduction in photothermal conversion efficiency at elevated salinity may result from the combined effects of altered thermophysical properties and salinity-induced aggregation. Future studies involving zeta potential measurements, particle-size analysis, and long-term stability evaluation are required to further clarify the role of aggregation in Fe_3_O_4_–NaCl nanofluid systems.

#### 3.4.4 Model comparison and recommendation of practical operating conditions

Table 6 summarizes the LOOCV performance of the predictive models. The RF model demonstrated superior predictive capability compared with the quadratic polynomial model, achieving a higher LOOCV R^2^ value and lower prediction errors. In addition to improved prediction accuracy, the RF model provided further insight into feature importance and nonlinear relationships between operating variables and *η*_nf_, which cannot be directly obtained from conventional regression-based approaches. Similar advantages of machine-learning approaches have been reported in recent studies on ferro-nanofluid flow, magnetohydrodynamic heat-transfer systems, and nanofluid-based battery cooling applications, where nonlinear interactions among operating variables were successfully captured using data-driven models (Ibrahim et al., 2026; Sharma et al., 2026; Shukla et al., 2026). From a practical standpoint, the RF model can assist in identifying favorable operating conditions and reducing experimental workload.

**TABLE 6:** Comparison of LOOCV performance for predictive models of saline nanofluid photothermal conversion efficiency.

Model	*R* ^2^	RMSE	MAE
Quadratic polynomial model (ridge regularization)	0.663	0.0443	0.0384
Random forest (RF)	0.757	0.0377	0.0301

Considering that saline conditions are unavoidable in practical applications such as solar seawater desalination and salt-gradient solar ponds, the model prediction results suggest that relatively high *η*_nf_ values can still be maintained under moderate salinity conditions. Although the highest predicted *η*_nf_ was obtained under zero-salinity conditions at a relatively low nanoparticle mass fraction, the range of *x*_2_ = 2–4 g L^-1^ combined with *x*_1_ = 0.05–0.10% may be regarded as a practical operating region that balances saline adaptability and photothermal conversion performance.

## 4 Conclusion

1. Salt concentration was identified as the dominant factor governing the photothermal conversion efficiency of Fe_3_O_4_/NaCl saline nanofluids (ANOVA, *p* < 0.001, partial *η* ^2^ = 0.769), whereas nanoparticle mass fraction within the investigated range (0%–0.20%) showed no statistically significant effect (*p* = 0.586). Tukey’s HSD test further confirmed significant differences in *η*_nf_ between low- and high-salinity conditions.
2. Both the quadratic polynomial model with Ridge regularization and the random forest model demonstrated acceptable predictive capability for *η*_nf_ prediction under limited-sample conditions. The optimized RF model achieved an LOOCV performance of *R* ^2^ = 0.757, RMSE = 0.0377, and MAE = 0.0301, outperforming the quadratic polynomial model (*R* ^2^ = 0.663, RMSE = 0.0443, and MAE = 0.0384). Furthermore, the RF model provided useful insight into the relative importance of salinity-related and nanoparticle-related descriptors as well as the nonlinear relationships governing *η*_nf_.
3. Model prediction results indicated that the highest predicted *η*_nf_ was obtained under zero-salinity conditions at a relatively low nanoparticle mass fraction. However, considering that saline environments are unavoidable in practical applications, relatively high *η*_nf_ values could still be maintained under moderate salinity conditions. Therefore, the range of *x*_2_ = 2–4 g L^−1^ combined with *x*_1_ = 0.05–0.10% may be regarded as a practical operating region balancing saline adaptability and photothermal conversion performance.

A limitation of the present study is that nanoparticle aggregation behavior under different salinity conditions was not directly characterized. Future work should combine zeta potential measurements, particle-size analysis, and long-term stability evaluation to further clarify the influence of salinity-induced aggregation on the photothermal performance of Fe_3_O_4_ saline nanofluids.

## Data Availability

The original contributions presented in the study are included in the article/Supplementary Material, further inquiries can be directed to the corresponding author.

## Supplementary material

Supplementary Figure S1 Morphological characterization of Fe_3_O_4_ nanoparticles: **(a)** SEM image; **(b)** TEM image; **(c)** particle size distribution.

Supplementary Figure S2 Heating and cooling curves of saline nanofluids under different nanoparticle mass fractions and salt concentrations. **(a)** 0 g/L NaCl; **(b)** 2 g/L NaCl; **(c)** 4 g/L NaCl; **(d)** 6 g/L NaCl; **(e)** 8 g/L NaCl.
