# Supplementary material for: Photothermal conversion efficiency of saline nanofluids: coupled effects of Fe3O4 nanoparticle mass fraction and salinity revealed by response surface and random forest modeling
Source: Front Chem. 2026 Jul 13;14:1890307. doi: 10.3389/fchem.2026.1890307 (PMC13402542; doi:10.3389/fchem.2026.1890307)
Supplement: Supplementary file 1 [file Supplementaryfile1.doc]

**Supporting information**

**Photothermal conversion efficiency of saline nanofluids: Coupled effects of Fe₃O₄ nanoparticle mass fraction and salinity revealed by response surface and random forest modeling**

Xiaojun Guo ^a,b^, Jia Liu ^a^, Zhiming Wu ^a^, Longjie Wang ^a^, Da Lin ^c^, Wenfeng Xiao ^c^, Xiao Song ^c^, Qianzhuo Zhu ^c^, Yuanrui Zhao ^c^, Yichuan Luo ^c^, Zhuangzhuang Jia c[[1]](#footnote-2)*

^a^ College of Mechanical and Electronic Engineering, Tarim University, Alar 843300, China

^b^ College of Engineering, China Agricultural University, Beijing 100083, China

^c^ College of Hydraulic and Architectural Engineering, Tarim University, Alar 843300, China

# 1. Characterization of Fe₃O₄ nanoparticles

The morphology and particle size distribution of Fe₃O₄ nanoparticles are presented in Figure S1.


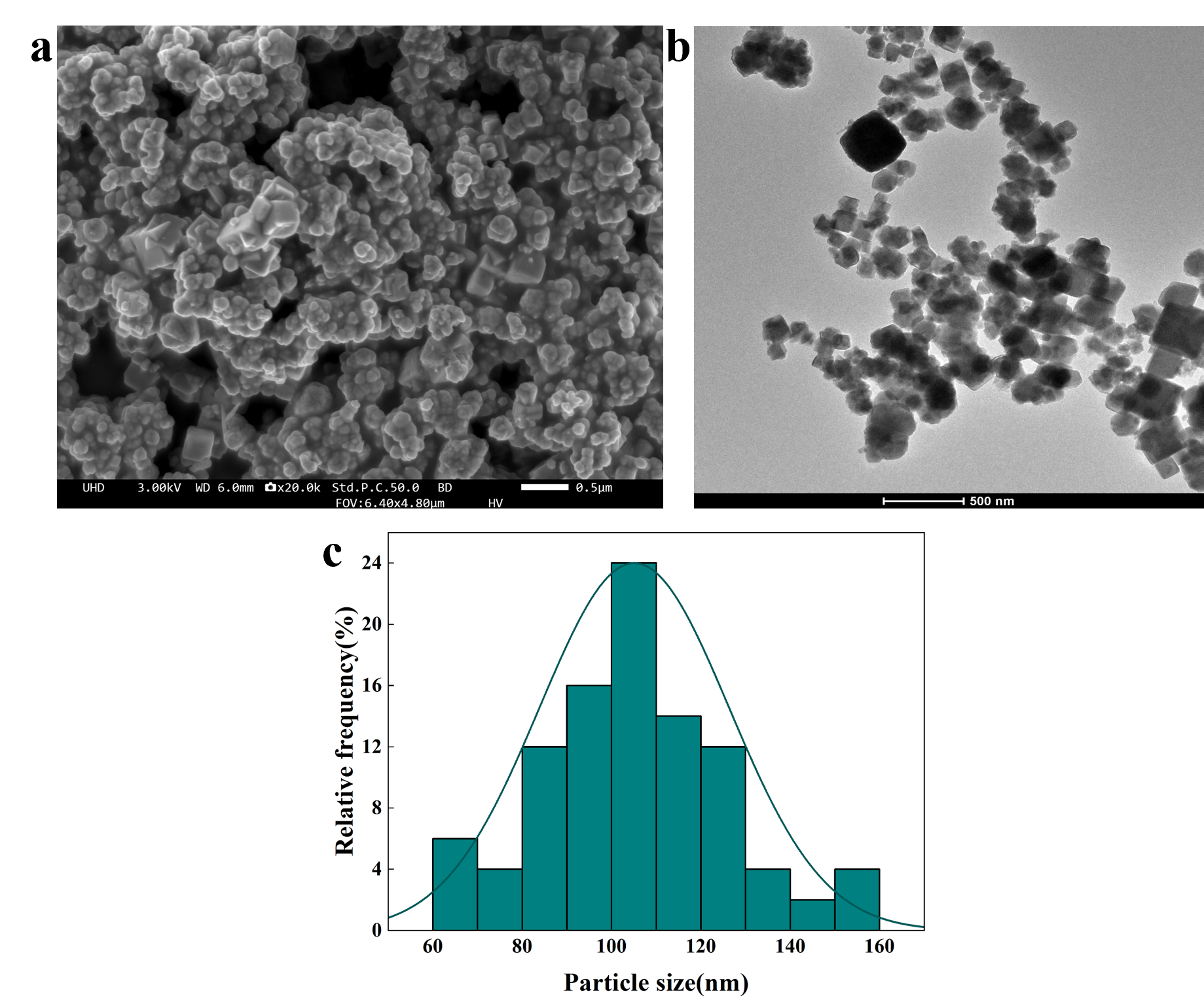


**Figure S1.** Morphological characterization of Fe₃O₄ nanoparticles: (a) SEM image; (b) TEM image; (c) particle size distribution.

# 2. Temperature evolution of saline nanofluids

Representative heating and cooling curves of saline nanofluids with different salt concentrations and nanoparticle mass fractions are shown in Figure S2.


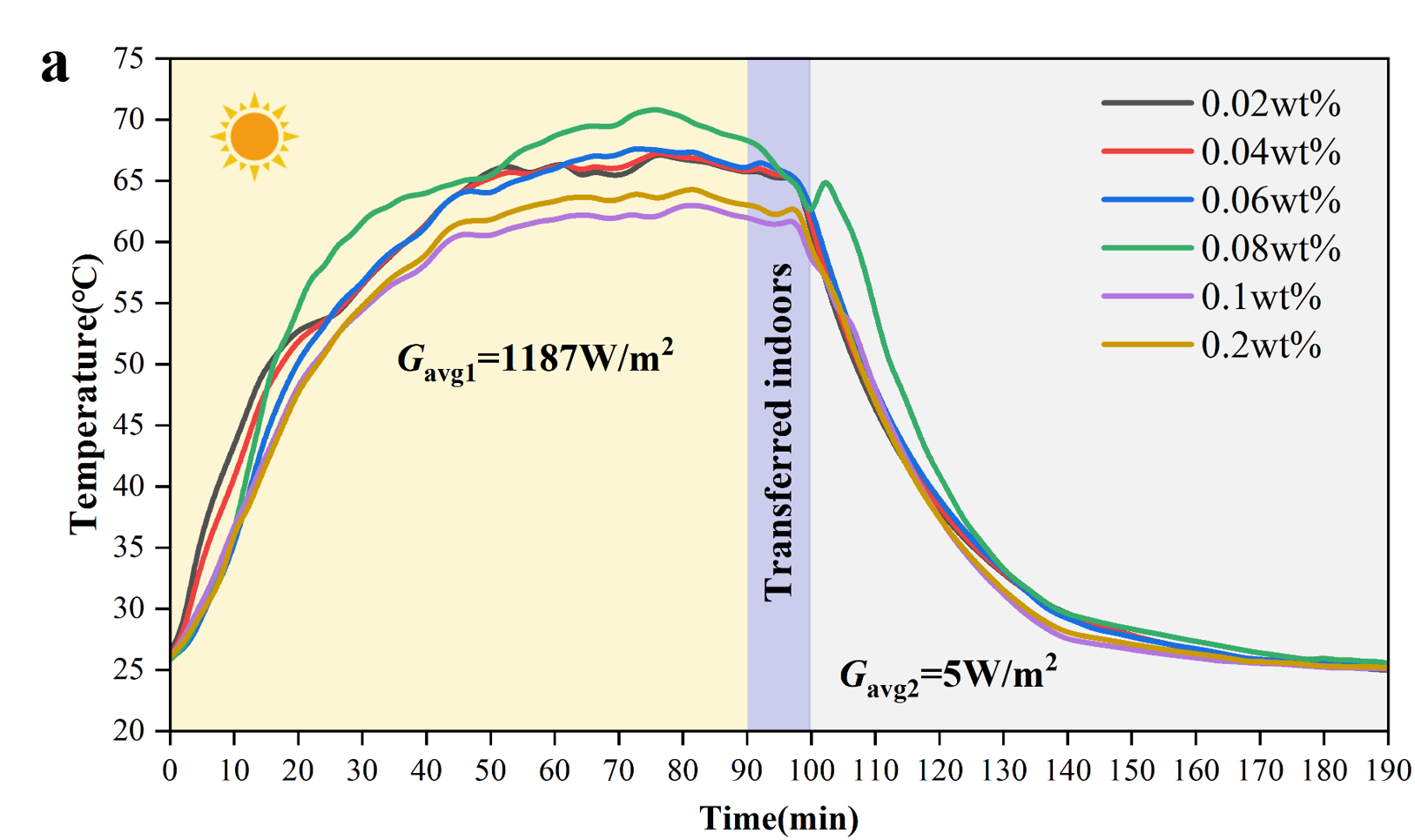


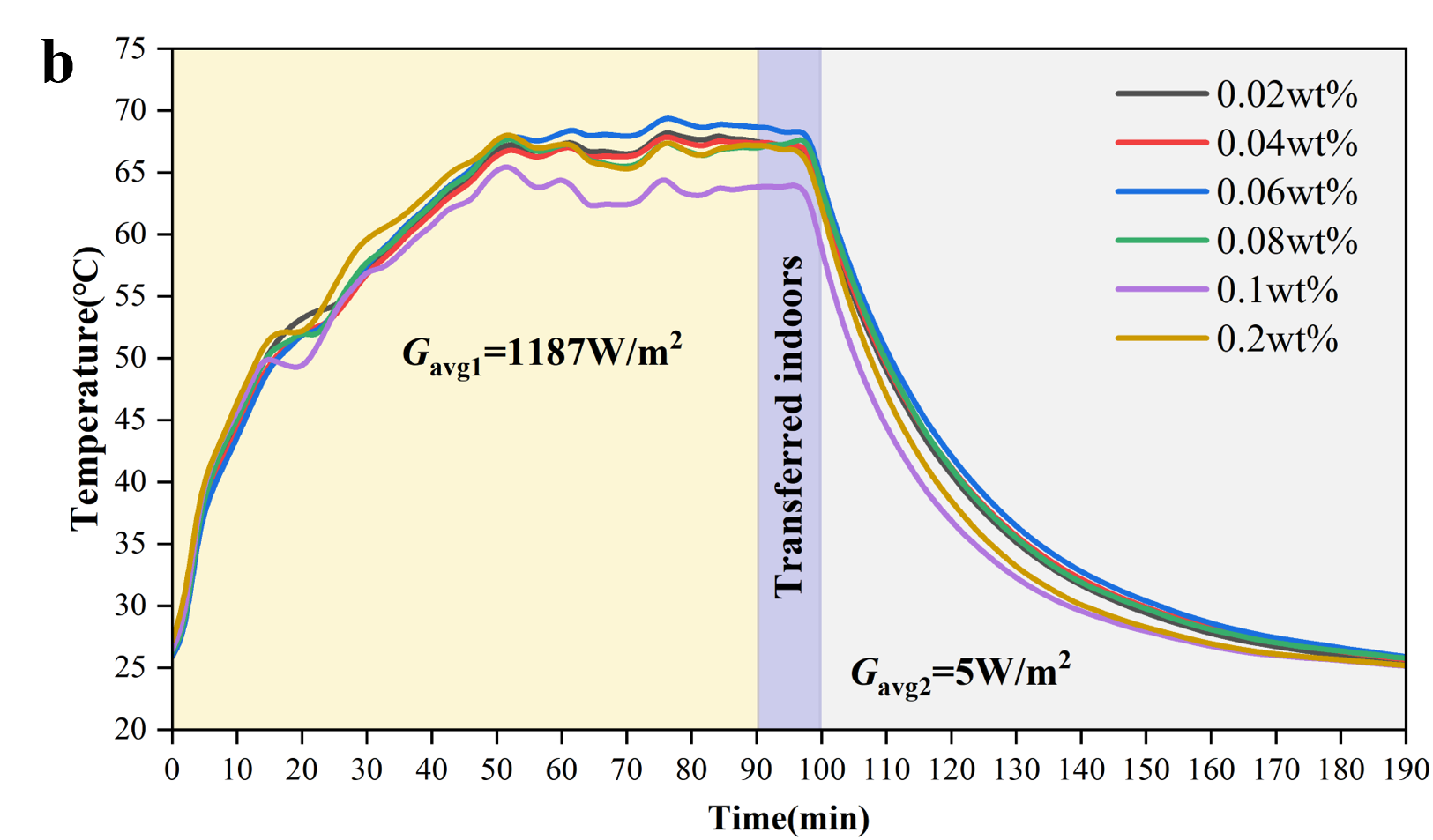


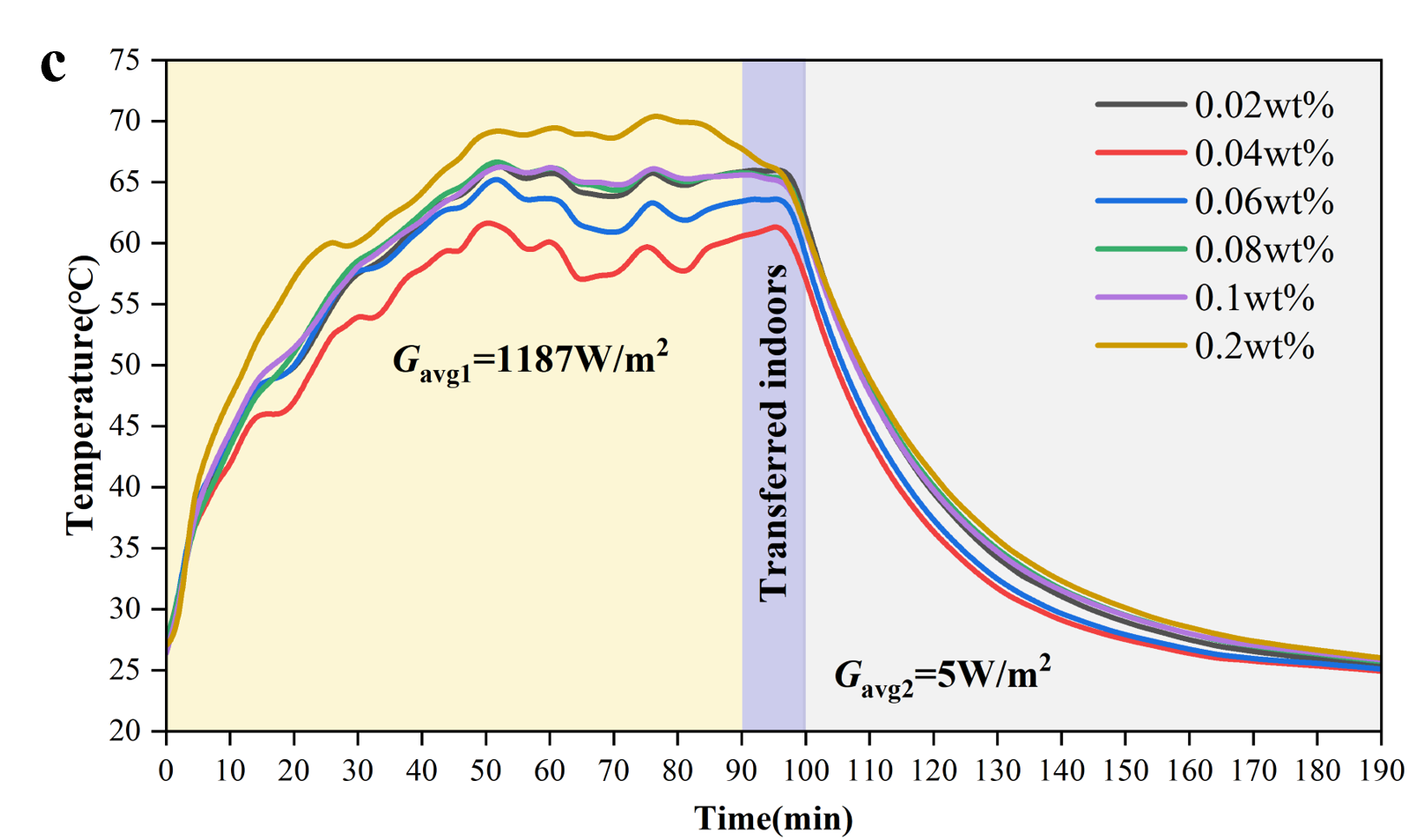


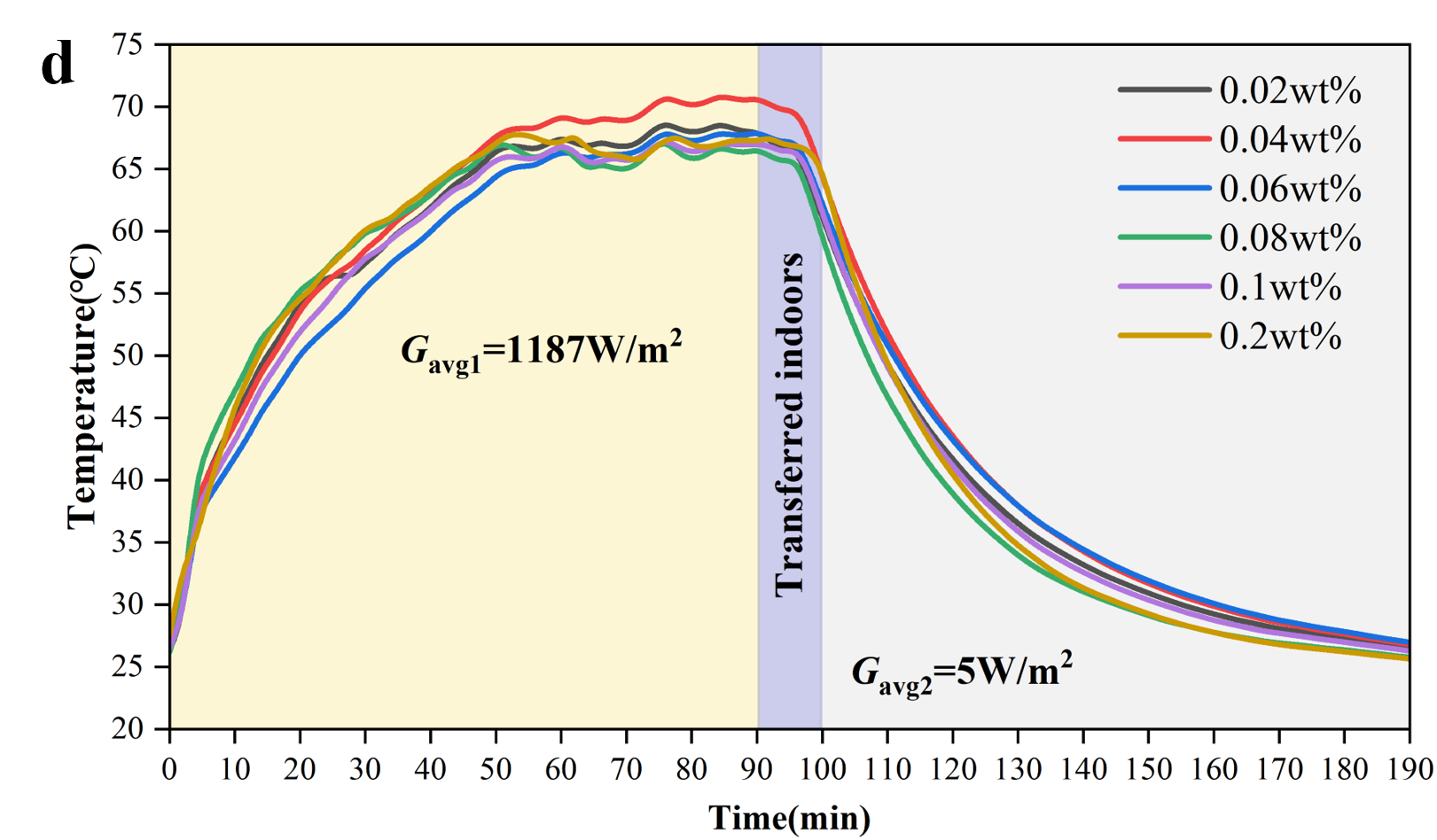


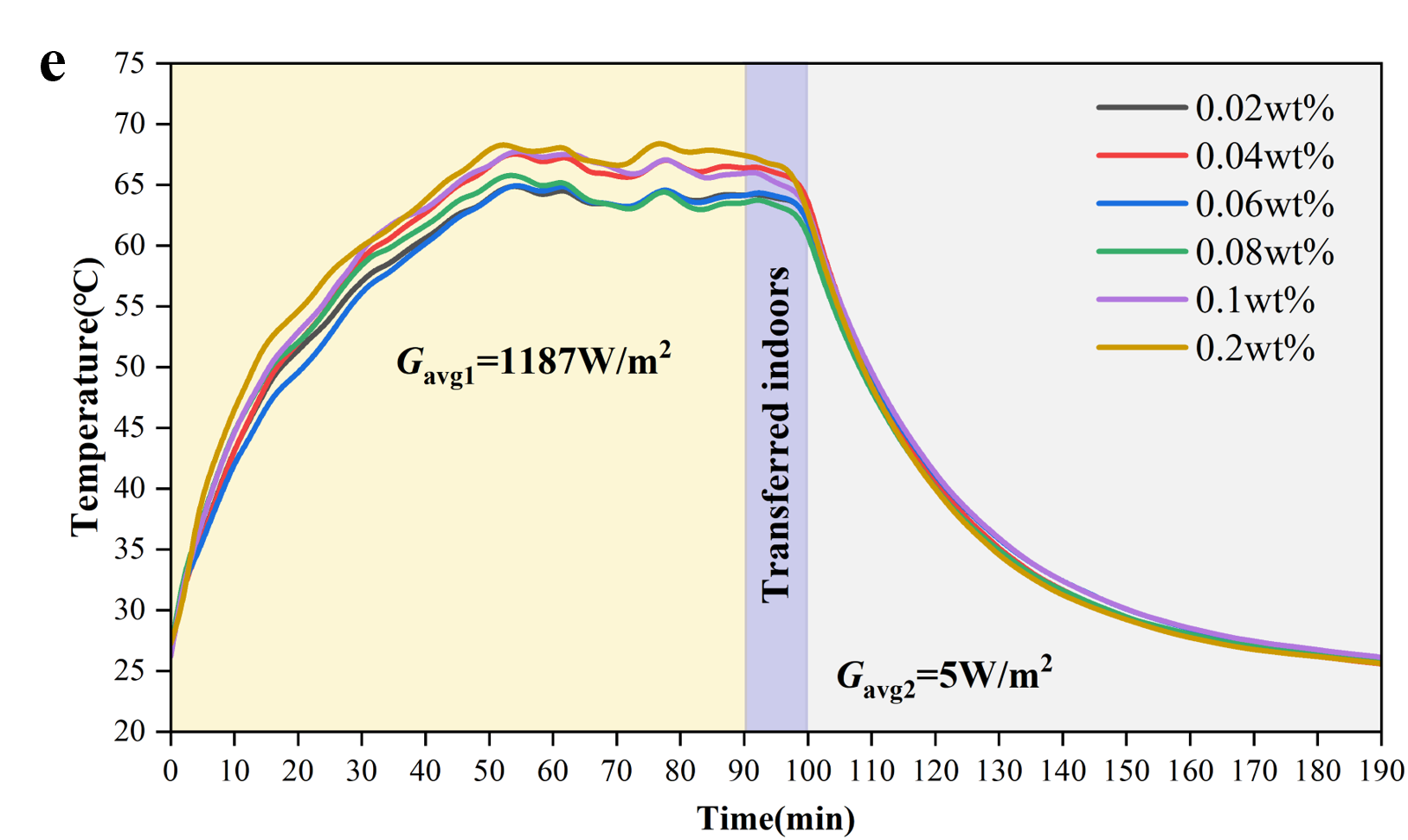


**Figure S2.** Heating and cooling curves of saline nanofluids under different nanoparticle mass fractions and salt concentrations. (a) 0 g/L NaCl; (b) 2 g/L NaCl; (c) 4 g/L NaCl; (d) 6 g/L NaCl; (e) 8 g/L NaCl.

1. ^^ Corresponding author: e-mail: jzz@taru.edu.cn [↑](#footnote-ref-2)
